# PRL-3和RhoC在A549细胞中的表达及意义

**DOI:** 10.3779/j.issn.1009-3419.2010.12.15

**Published:** 2010-12-20

**Authors:** 平 张, 志培 张, 香敏 李, 杰 雷, 凯 苏, 小飞 李, 小平 王

**Affiliations:** 1 710038 西安，第四军医大学唐都医院胸外科 Department of Toracic Surgery, Tangdu Hospital, the Fourth Military Medical University, Xi'an 710038, China; 2 810001 西宁，青海大学附属医院肾内科 Department of Nephrology, Qinghai University Afliated Hospital, Xi'ning 810001, China

**Keywords:** PRL-3, 细胞划痕实验, 细胞培养, Phosphatase of regenerating liver-3, Wound healing assay, Cell culture

## Abstract

**背景与目的:**

在前期研究中，我们发现在非小细胞肺癌中PRL-3和RhoC的表达具有相关性，提示两者可能存在相互作用。本研究通过检测两者在A549细胞迁移中的作用以及是否存在相互作用，为进一步研究它们在肿瘤发生发展中的作用机理提供实验依据。

**方法:**

应用PRL-3抗体、RhoC抗体分别阻断A549细胞中PRL-3和RhoC的功能，采用细胞划痕实验检测两者对其迁移能力的影响；应用RT-PCR检测PRL-3和RhoC表达的变化。

**结果:**

PRL-3和RhoC功能被阻断后，A549细胞迁移能力明显降低；阻断PRL-3的A549细胞中RhoC表达降低，然而阻断RhoC的A549细胞中PRL-3表达无明显改变。

**结论:**

PRL-3和RhoC可能具有促进A549细胞远处转移的功能；PRL-3可能具有上调RhoC表达的功能；PRL-3促进癌细胞远处转移的功能可能是通过RhoC及其下游因子实现的，而RhoC对PRL-3的表达可能无反馈性调节作用。

## Abstract (en)

**Background and objective:**

The expression of phosphatase of regenerating liver-3 (PRL-3) is cor- related with Ras homologue C (RhoC) in non-small cell lung cancer (NSCLC), suggesting that they have interactions. The aim of this study is to investigate the functions of PRL-3 and RhoC in the migration of A549 cell and the potential mechanism of PRL-3 and RhoC in carcinogenesis and cancer development.

**Methods:**

PRL-3Ab and RhoCAb were used to block the functions of PRL-3 and RhoC respectively. Wound healing assay was applied to detect the migration of A549 cell and the ex- pression levels of PRL-3 and RhoC were detected by RT-PCR.

**Results:**

The migration of A549 cell decreased after blockage of PRL-3 and RhoC. The expression of RhoC decreased when PRL-3 was blocked without any changes on the expression of PRL-3.

**Conclusion:**

PRL-3, RhoC could increase cell migration in A549 cells.

肺癌是一种严重威胁人类健康的恶性肿瘤，其发病率和死亡率呈上升趋势，因此急需对这一疾病进行深入的研究。近年来的研究发现，磷酸酶家族中的肝再生磷酸酶（phosphatase of regenerating liver, PRL）亚家族包括PRL1-3三种分子，其成员PRL-3高表达在结直肠癌^[1, 2]^、卵巢癌^[3]^和胃癌^[4]^等肿瘤细胞的增殖、粘附、迁移、侵袭以及转移中发挥重要的正向调控作用，但有关PRL -3在肺癌中的作用机制仍不十分清楚；RhoC属于小分子G蛋白超家族中的Rho亚家族，是Rho信号转导通路的重要分子，在细胞的信号转导通路中作为信号转换器，调控各种细胞骨架运动、细胞形态建成等功能^[5]^，并可通过对细胞骨架蛋白的调节加快细胞迁移^[6, 7]^。许多研究^[8-10]^表明RhoC在多种肿瘤中高表达并与肿瘤的浸润转移高度相关，但是在该通路中RhoC蛋白活化及其信号传递机制尚不清楚。Fiordalisi等^[11]^通过体外实验提示PRL-3可能是通过激活Rho信号转导通路来促进肿瘤的运动与转移。本研究应用抗体分别阻断A549细胞中PRL-3和RhoC的功能，采用细胞划痕实验检测两者对其迁移能力的影响，并应用RT-PCR检测PRL-3和RhoC表达的变化，为进一步研究它们在肿瘤发生发展中的作用机理提供实验依据。

## 1 材料和方法

### 1.1 材料与试剂

A549细胞由第四军医大学唐都医院胸外科实验室提供，鼠抗人PRL-3单克隆抗体为Santa Cruz公司产品，兔抗人RhoC多克隆抗体为北京博奥森生物技术有限公司产品，新生牛血清为兰州荣晔生物科技有限责任公司产品，DMEM培养液为Gibco产品，RNAiso Plus、反转录试剂盒、PCR试剂盒均为TaKaRa产品。

### 1.2 细胞划痕实验

在96孔板背面用Marker通过孔的直径画标记线，将生长状态良好的A549细胞接种于96孔板，每孔约8 000个细胞，细胞长满后吸出培养液，用200 μL Tip头垂直于标记线划痕，PBS液冲洗3次；第1排加入含1:400抗人PRL-3抗体的5%血清培养液，第2排加入含1:400抗人RhoC抗体的5%血清培养液，第3排加入不含抗体的5%血清培养液作对照。分别于0 h、6 h、12 h、24 h照相。

### 1.3 RT-PCR

将细胞划痕实验中所用的三组A549细胞分别接种至培养瓶中，每组8瓶，共24瓶，继续应用含1:400抗人PRL-3抗体、含1:400抗人RhoC抗体以及不含抗体的5%新生牛血清DMEM培养液分别培养，细胞长满85%后用RNAiso Plus提取总RNA，反转录后进行PCR检测三组细胞中PRL-3和RhoC的表达情况。反应条件：β-actin：95 ℃、5 min；94 ℃、30 s，54 ℃、40 s，72 ℃、1 min，35个循环；72 ℃、5 min；4 ℃保存产物。PRL -3：95 ℃、5 min；94 ℃、30 s，61 ℃、40 s，72 ℃、1 min，35个循环；72 ℃、5 min；4 ℃保存产物。RhoC：95 ℃、5 min；94 ℃、30 s，57 ℃、40 s，72 ℃、1 min，35个循环；72 ℃、5 min；4 ℃保存产物。所用引物序列、目的片段长度、退火温度见表 1。应用AlphaImager系统中的SpotDenso分析工具测定RT-PCR各个目标条带的光密度值（isodensity value, IDV）值。

**1:** *β-actin*、*PRL-3*、*RhoC*基因引物序列 Primer sequences of *β-actin*, *PRL-3* and *RhoC* genes

Gene	Primer sequences	Product length	Tempreture
β-actin	F: 5’-AGCGAGCATCCCCCAAAGTT-3’ R: 5’-GGGCACGAAGGCTCATCATT-3’	285bp	54 ℃
PRL-3	F: 5’-ATGGCTCGGATGAACCGCCCGGC-3’	481 bp	61 ℃
	R: 5’-TGAACCGCAGCCTATGTTTGGGC-3’		
RhoC	F: 5’-TCCTCATCGTCTTCAGCAAG-3’	182 bp	57 ℃
	R: 5’-GAGGATGACATCAGTGTCCT-3’		

### 1.4 统计学分析

应用SPSS 12.0统计分析软件，使用*Paired Samples t Test*分析RT-PCR结果，*P* < 0.05为差异有统计学意义。

## 2 结果

### 2.1 细胞划痕实验结果

0 h时观察：三组细胞划痕良好，划痕宽度基本一致；6 h时观察：对照组细胞向划痕处爬行明显，加PRL-3抗体、RhoC抗体的两组细胞迁移不明显；12 h时观察：对照组细胞已爬行至划痕中心，加PRL-3抗体、RhoC抗体的两组细胞划痕宽度较0 h明显变窄，两组之间无明显差别；24 h时观察：对照组细胞已基本爬满划痕，加PRL-3抗体、RhoC抗体的两组细胞仍未爬行至划痕中心，两组之间无明显差别。培养液中加抗体的细胞的迁移速度明显比培养液中不加抗体的细胞慢，加PRL-3抗体、RhoC抗体的两组细胞迁移速度无明显差别（图 1，图中粗线为Marker标记）。

**1:**
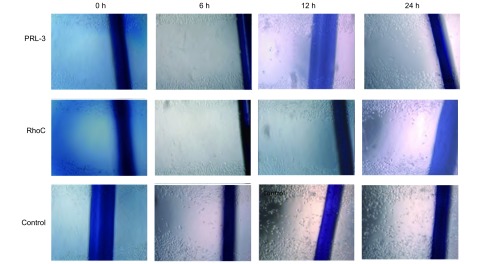
细胞划痕实验 Wound healing assays

### 2.2 RT-PCR结果

加RhoC抗体组PRL -3表达的IDV值为（21 509 514.75±1 100 223.540），对照组PRL-3表达的IDV值为（21 826 848.25±877 815.062），两组间差异无统计学意义（*t*=2.047, *P*=0.08）；加PRL-3抗体组RhoC表达的IDV值为（38 303 080.13±1 907 724.847），对照组RhoC的表达的IDV值为（56 822 463.00±2 690 278.654），两组间差异有统计学意义（*t*=19.032, *P* < 0.001）（图 2）。PRL-3的表达在加RhoC抗体组与对照组之间差异无统计学意义（*P*=0.08），RhoC的表达在加PRL-3抗体组与对照组之间差异有统计学意义（*P* < 0.001）。

**2:**
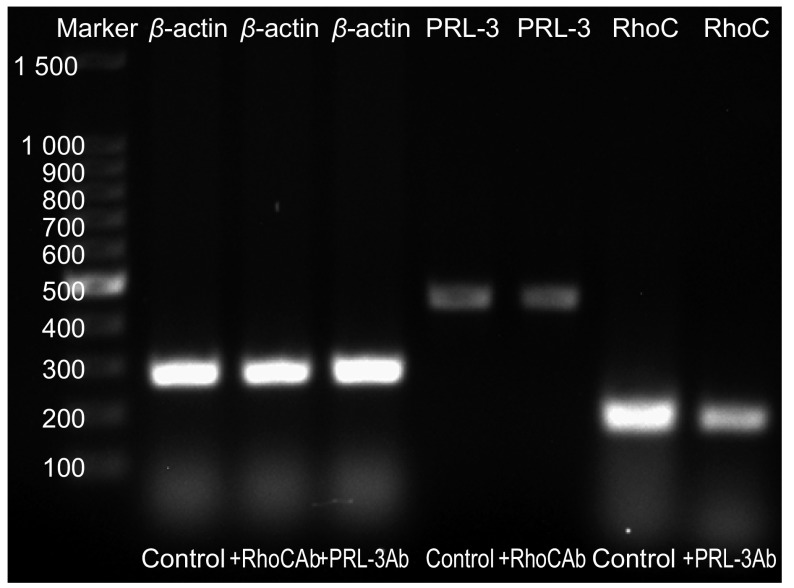
*β-actin*、*PRL-3*、*RhoC*基因RT-PCR扩增产物凝胶电泳成像 Gel electrophoresis photographs of RT- PCR products amplified for *β-actin*, *PRL-3* and *RhoC* gene

## 3 讨论

人类*PRL-3*基因位于染色体8q24.3，编码173个氨基酸，其分子质量约为20 kDa，属于非典型磷酸酪氨酸蛋白磷酸脂酶，核磁共振分析表明PRL -3以单体形式存在，其二级结构由5个β折叠和6个α螺旋组成，它们的排列和整体折叠方式为双特异性磷酸酶（dual-specificity phosphatase, DSP）所特有。具有催化活性的Cys104、Arg110和它们之间的几个疏水性氨基酸组成的P-环构成了PRL-3的磷酸酶活性中心，后者可能决定着PRL-3底物的特异性，使得PRL-3有别于其它双特异磷酸酶。72位的Asp则起到一个广义酸的作用，其所在的噜噗环结构独立于蛋白的其它结构域，在PRL-3和底物结合时可以发生空间结构的变化，对于PRL-3磷酸酶活性的发挥具有重要作用^[12, 13]^，主要在心肌、平滑肌和骨骼肌等正常组织中表达^[14]^。Saha等^[1]^发现PRL-3在结直肠癌转移组织中过度表达，在正常结、直肠粘膜和原位癌中低度表达，在伴转移的原发癌中中度表达，而且在随访中还发现原发癌切除术后出现远处转移的几率随原发癌组织中PRL-3表达强度的增高而增加，这一发现提示PRL-3可能有促进肿瘤细胞远处转移的功能。Fiordalisi等^[15]^应用异戊二烯转移酶抑制剂FTI-2153处理转染了PRL-3的SW480细胞后，PRL-3由胞浆的膜结构转移到细胞核中，并且丧失了促进细胞浸润和迁移的能力。Dursina等^[16]^成功筛选得到了两个化合物，对PRL-3等蛋白的异戊二烯化修饰具有较强的抑制作用，这些研究为更深入研究PRL-3的功能奠定了基础。本实验通过对比分析阻断PRL-3功能前后A549细胞迁移能力的变化，发现阻断PRL-3功能后A549细胞的迁移能力明显减慢，这一现象再次提示PRL-3可能有促进肿瘤细胞远处转移的功能。

*RhoC*基因定位于1p13-p21，其编码蛋白含193个氨基酸，属于Ras超家族中的小GTP结合蛋白亚家族，在真核细胞中作为分子开关控制着众多信号转导途径^[17]^。R h℃蛋白具有调控细胞骨架运动、细胞形态建成等功能^[5]^，并可通过对细胞骨架蛋白的调节促进细胞迁移^[6, 7]^。在该通路中，RhoC蛋白的活化及其调控机制尚不清楚。在本实验中应用抗体阻断RhoC功能，研究A549细胞迁移能力的变化，发现阻断RhoC功能后A549细胞的迁移能力明显减慢，再次证实RhoC可能有加快细胞迁移的功能。

在前期研究中，我们应用免疫组织化学方法检测了92例人非小细胞肺癌组织标本，发现在非小细胞肺癌中PRL-3和RhoC的表达具有相关性，提示两者可能存在相互作用；同时在转移能力较强的人非小细胞肺癌组织标本中PRL-3和RhoC蛋白的表达均较高，两者可能具有促进癌细胞远处转移的生物学功能^[18]^。在这一发现的提示下我们做了细胞划痕实验，发现应用抗体阻断PRL -3和RhoC功能后，A549细胞迁移能力降低，这一现象提示PRL-3和RhoC可能有促进肿瘤细胞远处转移的功能，这一现象再次证实了上述结论。我们应用RT-PCR检测阻断两者后A549细胞中RhoC和PRL-3表达的变化，发现培养液中加PRL-3抗体的A549细胞中RhoC的表达降低，提示PRL-3可能通过某些环节上调RhoC的表达，PRL-3可能通过RhoC及其下游因子促进癌细胞的远处转移。目前RhoC蛋白比较明确的功能是引起F-肌动蛋白的重组和动态变化^[19]^，但是在该通路中RhoC蛋白活化及其信号传递机制尚不清楚，本实验结果提示PRL -3可能是该通路中RhoC蛋白的上游信号分子，通过上调RhoC蛋白的表达促进肿瘤细胞远处转移；但PRL-3的活化及其信号传递机制并不十分清楚，有关PRL-3信号通路上游分子的研究可能会给肿瘤发生发展机制的研究打开一个新的视野。在本实验中还发现加RhoC抗体的A549细胞中PRL-3的表达无明显改变，提示RhoC对PRL-3的表达可能无反馈性调节作用，PRL-3和RhoC所在信号通路的负反馈性调节功能可能通过其它分子执行，这方面的研究还需进一步深入。
